# Mechanical ventilation characteristics and their prediction performance for the risk of moderate and severe bronchopulmonary dysplasia in infants with gestational age <30 weeks and birth weight <1,500 g

**DOI:** 10.3389/fped.2022.993167

**Published:** 2022-11-02

**Authors:** Jing Yin, Linjie Liu, Huimin Li, Xuewen Hou, Jingjing Chen, Shuping Han, Xiaohui Chen

**Affiliations:** Department of Paediatrics, Women’s Hospital of Nanjing Medical University, Nanjing Maternity and Child Health Care Hospital, Nanjing, China

**Keywords:** mechanical ventilation, bronchopulmonary dysplasia, preterm infants, predictive model, newborn

## Abstract

**Introduction:**

Moderate and severe bronchopulmonary dysplasia (BPD) is a common pulmonary complication in premature infants, which seriously affects their survival rate and quality of life. This study aimed to describe the mechanical ventilation characteristics and evaluate their prediction performance for the risk of moderate and severe BPD in infants with gestational age <30 weeks and birth weight <1,500 g on postnatal Day 14.

**Methods:**

In this retrospective cohort study, 412 infants with gestational age <30 weeks and birth weight <1,500 g were included in the analysis, including 104 infants with moderate and severe BPD and 308 infants without moderate and severe BPD (as controls). LASSO regression was used to optimize variable selection, and Logistic regression was applied to build a predictive model. Nomograms were developed visually using the selected variables. To validate the model, receiver operating characteristic (ROC) curve, calibration plot, and clinical impact curve were used.

**Results:**

From the original 28 variables studied, six predictors, namely birth weight, 5 min apgar score, neonatal respiratory distress syndrome (≥Class II), neonatal pneumonia, duration of invasive mechanical ventilation (IMV) and maximum of FiO_2_ (fraction of inspiration O_2_) were identified by LASSO regression analysis. The model constructed using these six predictors and a proven risk factor (gestational age) displayed good prediction performance for moderate and severe BPD, with an area under the ROC of 0.917 (sensitivity = 0.897, specificity = 0.797) in the training set and 0.931 (sensitivity = 0.885, specificity = 0.844) in the validation set, and was well calibrated (*P*_Hosmer-Lemeshow test _= 0.727 and 0.809 for the training and validation set, respectively).

**Conclusion:**

The model included gestational age, birth weight, 5 min apgar score, neonatal respiratory distress syndrome (≥Class II), neonatal pneumonia, duration of IMV and maximum of FiO_2_ had good prediction performance for predicting moderate and severe BPD in infants with gestational age <30 weeks and birth weight <1,500 g on postnatal Day 14.

## Introduction

In preterm infants, bronchopulmonary dysplasia (BPD) is one of the most common and serious pulmonary complications, seriously affecting the quality of their lives. The definition of BPD is oxygen support as needed for at least 28 days (1). According to their required fraction of inspired oxygen (FiO_2_) at 36 weeks of corrected gestational age, infants with gestational age <32 weeks were categorized as mild (no oxygen requirement), moderate (21%–30%) or severe BPD (over 30% or positive pressure assistance) (1). With the improvement of the treatment success rate of very low birth weight infants (VLBWI, birth weight <1,500 g) and extremely low birth weight infants (ELBWI, birth weight <1,000 g), the incidence of BPD is also increasing. According to the studies from many countries, BPD’s incidence varies from 11 to 50%, due to the different diagnostic and treatment criteria (2). BPD incidence increases as the gestational age or birth weight decreases. Previous studies have reported that about 30% of VLBWI suffer from BPD (3), the incidence rate of BPD fluctuated between 12.9% and 41% in infants with gestational age <32 weeks, and the incidence rate can reach 80% in infants with gestational age <25 weeks (4). The quality of life for preterm infants with BPD is reduced due to a higher mortality rate and a higher incidence of pulmonary, cardiovascular, and neurodevelopmental disorders (5, 6). Moreover, preterm infants with moderate and severe BPD are more likely to suffer complications and comorbidities, including longer hospital stays, respiratory support after discharge and higher death risk (7, 8). Therefore, clarifying the predictors of moderate and severe BPD, early screening and prevention of moderate and severe BPD is of great significance not only for clinical control of moderate and severe BPD, but also for improving the prognosis of preterm infants.

Many clinical risk factors for BPD have been reported in infants with gestational age <32 weeks over the past few decades (9). To our knowledge, smaller gestational age and lower birth weight are proven risk factors for BPD (9). Other perinatal and postpartum factors can increase the risk of BPD, such as chorioamnionitis, patent ductus arteriosus (PDA), neonatal pneumonia, neonatal respiratory distress syndrome (10). Moreover, supportive care with mechanical ventilation is an essential strategy for managing severe neonatal respiratory failure. It is well known that the ventilator-induced lung injury is an important risk factor for BPD (11). In a study involving 17 centers, the authors developed and validated models for BPD risk at 6 postnatal ages using gestational age, birth weight, race and ethnicity, sex, respiratory support, and FiO_2_, and found that gestational age conveyed the most predictive information for BPD risk on Postnatal Days 1 and 3, and respiratory support on Days 7, 14, 21, and 28 (12). Although previous predictive models for BPD have been described (12–14), studies focused on the risk factors and prediction models for moderate and severe BPD are few, especially in infants with gestational age <30 weeks and birth weight <1,500 g. In the current study, based on the clinical characteristics and the different types of mechanical ventilation up to postnatal Day 14, we analysed and identified risk factors affecting moderate and severe BPD, and developed a meaningful risk prediction model for paediatricians and neonatologists to perform early screening of moderate and severe BPD in infants with gestational age <30 weeks and birth weight <1,500 g on postnatal Day 14. The prediction model could be used to target VLBWI at the highest risk of moderate and severe BPD, to tailor follow-up and preventive measures for VLBWI.

## Methods

### Participants and data collection

This was a retrospective study based on electronic medical records from NingBX neonatal perinatal network (http://www.ningbx.com). Infants with gestational age <30 weeks and birth weight <1,500 g were recruited at the Department of Pediatrics, Nanjing Maternity and Child Health Care Hospital from January 2018 to December 2021. This study was approved by the Ethics Committee of Nanjing Maternity and Child Health Care Hospital, and either a legal guardian or parent provided informed consent. Moderate and severe BPD is defined as oxygen requirement (FiO_2 _> 21%) for at least 28 days, and the need for any type of respiratory support at 36 weeks post-menstrual age (1, 15). The preterm infants with moderate and severe BPD were assigned to the case group, while preterm infants without moderate and severe BPD were assigned to the control group. Inclusion criteria were: (1) gestational age at birth <30 weeks and birth weight <1,500 g; (2) infants hospitalized within 1 day after birth. Whereas exclusion criteria were: (1) infants hospitalized for less than 28 days and lost to follow-up after discharge, who still required continued oxygen inhalation and hospitalization; (2) infants with severe congenital malformation; (3) infants with pneumothorax or pleural effusion; (4) infants undergoing surgery; (5) infants who died less than 28 days after birth; (6) incomplete data. At last, 412 infants with gestational age <30 weeks and birth weight <1,500 g were included in the analysis, including 104 infants with moderate and severe BPD and 308 controls.

We also collected multiple maternal and neonatal data according to electronic medical records up to postnatal Day 14, including maternal characteristics, medication use, neonatal characteristics, mechanical ventilation and oxygen requirement (Table 1). The maternal characteristics included gestational diabetes mellitus, maternal hypertension and chorioamnionitis. Medication use included prenatal use of glucocorticoids and magnesium sulfate. The neonatal characteristics included infant gender, gestational age at birth (weeks), birth weight (g), delivery mode, 1 min and 5 min apgar score, neonatal asphyxia, neonatal respiratory distress syndrome (≥Class II), neonatal pneumonia, patent ductus arteriosus (PDA), PDA size (mm), PDA treatment, PDA and ventricular septal defect, intraventricular hemorrhage (IVH III/IV), sepsis, and necrotizing enterocolitis (NEC ≥ Stage II). Neonatal asphyxia is defined as the inability of neonates to initiate and maintain breathing at birth, followed by impaired gas exchange, resulting in progressive hypoxemia, hypercapnia, and severe metabolic acidosis. Mild asphyxia is diagnosed by 1 min apgar score ≤7, or 5 min apgar score ≤7 and umbilical cord arterial pH <7.2; whereas severe asphyxia is diagnosed by 1 min apgar score ≤3, or 5 min apgar score ≤5 and umbilical cord arterial pH <7.0.

**Table 1:** Characteristics of the 412 preterm infants with gestational age <30 weeks and birth weight <1,500 g enrolled in the study according to presence/absence of moderate and severe bronchopulmonary dysplasia (BPD) and randomization to training set and validation set.

Items	Total infant cohort (*n *= 412)	Infants with moderate and severe BPD (*n *= 104)	Infants without moderate and severe BPD (*n *= 308)	Training set (*n* = 309)	Validation set (*n* = 103)	*P* value
Boy	236 (57.3%)	60 (57.7%)	176 (57.1%)	134 (43.4%)	42 (40.8%)	0.922
Gestational age (weeks)	28.3 (27.1, 29.1)	27.1 (26.0, 28.1)	28.6 (27.9, 29.3)	28.3 (27.1, 29.0)	28.7 (27.4, 29.3)	<0.001
Birth weight (g)	1109.0 ± 200.9	961.7 ± 191.3	1158.8 ± 178.7	1098.4 ± 195.8	1140.9 ± 213.6	<0.001
Cesarean delivery	187 (45.4%)	27 (26.0%)	160 (51.9%)	137 (44.3%)	50 (48.5%)	<0.001
1 min apgar score	8 (6, 10)	6 (3.3, 8)	9 (7, 10)	8 (6, 10)	8 (6, 9)	<0.001
5 min apgar score	9 (8, 10)	8 (7.3, 9)	10 (9, 10)	9 (8, 10)	9 (8, 10)	<0.001
Gestational diabetes mellitus	86 (20.9%)	20 (19.2%)	66 (21.4%)	70 (22.7%)	16 (15.5%)	0.633
Maternal hypertension	32 (7.8%)	6 (5.8%)	26 (8.4%)	27 (8.7%)	5 (4.9%)	0.379
Chorioamnionitis	151 (36.7%)	31 (29.8%)	120 (39.0%)	119 (38.5%)	32 (31.1%)	0.094
Prenatal use of glucocorticoids	380 (92.2%)	94 (90.4%)	286 (92.9%)	291 (94.2%)	89 (86.4%)	0.415
Prenatal use of magnesium sulfate	270 (65.5%)	73 (70.2%)	197 (64.0%)	198 (64.1%)	72 (69.9%)	0.248
Neonatal asphyxia	144 (35.0%)	64 (61.5%)	80 (26.0%)	111 (35.9%)	33 (32.0%)	<0.001
Neonatal respiratory distress syndrome (≥Class II)	49 (11.9%)	25 (24.0%)	24 (7.8%)	35 (11.3%)	14 (13.6%)	<0.001
Neonatal pneumonia	159 (38.6%)	77 (74.0%)	82 (26.6%)	120 (38.8%)	39 (37.9%)	<0.001
Patent ductus arteriosus (PDA)	297 (72.1%)	84 (80.8%)	213 (69.2%)	234 (75.7%)	63 (61.2%)	0.022
PDA size (mm)	1.7 (0, 2.3)	2.0 (0, 2.6)	1.5 (0, 2.2)	1.7 (0.4, 2.3)	1.3 (0, 2.3)	0.002
PDA treatment	99 (24.0%)	55 (52.9%)	44 (14.3%)	74 (23.9%)	25 (24.3%)	<0.001
PDA and ventricular septal defect	6 (1.5%)	2 (1.9%)	4 (1.3%)	5 (1.6%)	1 (1.0%)	0.645
Intraventricular hemorrhage (IVH III/IV)	72 (17.5%)	35 (33.7%)	37 (12.0%)	57 (18.4%)	15 (14.6%)	<0.001
Sepsis	130 (31.6%)	46 (44.2%)	84 (27.3%)	102 (33.0%)	28 (27.2%)	0.001
Necrotizing enterocolitis (NEC ≥ Stage II)	25 (6.1%)	11 (10.6%)	14 (4.5%)	23 (7.4%)	2 (1.9%)	0.026
Conventional mechanical ventilation (CMV)	141 (34.2%)	69 (66.3%)	72 (23.4%)	112 (36.2%)	29 (28.2%)	<0.001
Duration of CMV (days)	0 (0, 3)	5 (0, 10)	0 (0, 0)	0 (0, 4)	0 (0, 1)	<0.001
High frequency ventilation (HFV)	36 (8.7%)	30 (28.8%)	6 (1.9%)	28 (9.1%)	8 (7.8%)	<0.001
Duration of HFV (days)	0 (0, 0)	0 (0, 1)	0 (0, 0)	0 (0, 0)	0 (0, 0)	<0.001
Continuous positive airway pressure (CPAP)	315 (76.5%)	45 (43.3%)	270 (87.7%)	240 (77.7%)	75 (72.8%)	<0.001
Duration of CPAP (days)	6 (1, 10)	0 (0, 4)	7.5 (4, 11)	6 (1, 11)	5 (0, 9)	<0.001
High-flow nasal cannula (HFNC)	161 (39.1%)	5 (4.8%)	156 (50.6%)	124 (40.1%)	37 (35.9%)	<0.001
Duration of HFNC (days)	0 (0, 3)	0 (0, 0)	1 (0, 4)	0 (0, 4)	0 (0, 3)	<0.001
Bi-level positive airway pressure (BiPAP)	29 (7.0%)	8 (7.7%)	21 (6.8%)	20 (6.5%)	9 (8.7%)	0.763
Duration of BIPAP (days)	0 (0, 0)	0 (0, 0)	0 (0, 0)	0 (0, 0)	0 (0, 0)	0.792
Nasal intermittent positive pressure ventilation (NIPPV)	170 (41.3%)	58 (55.8%)	112 (36.4%)	116 (37.5%)	54 (52.4%)	0.001
Duration of NIPPV (days)	0 (0, 5)	2 (0, 6.8)	0 (0, 4)	0 (0, 4)	1 (0, 7)	0.002
Invasive mechanical ventilation (IMV)	148 (35.9%)	76 (73.1%)	72 (23.4%)	114 (36.9%)	34 (33.0%)	<0.001
Duration of IMV (days)	0 (0, 4)	7 (0, 13)	0 (0, 0)	0 (0, 4.5)	0 (0, 3)	<0.001
Noninvasive mechanical ventilation (NIMV)	382 (92.7%)	79 (76.0%)	303 (98.4%)	285 (92.2%)	97 (94.2%)	<0.001
Duration of NIMV (days)	14 (10, 14)	7 (1, 14)	14 (12, 14)	14 (9, 14)	14 (11, 14)	<0.001
Duration of FiO_2 _> 21% (days)	14 (14, 14)	14 (14, 14)	14 (14, 14)	14 (14, 14)	14 (14, 14)	0.019
Maximum of FiO_2_ (%)	30 (30, 45)	50 (40, 80)	30 (25, 40)	30 (30, 45)	35 (30, 40)	<0.001

Note: *P* value for comparison between infants with and without moderate and severe BPD.

The characteristics of mechanical ventilation and oxygen requirement included conventional mechanical ventilation (CMV), duration of CMV (days), high frequency ventilation (HFV), duration of HFV (days), continuous positive airway pressure (CPAP), duration of CPAP (days), high-flow nasal cannula (HFNC), duration of HFNC (days), Bi-level positive airway pressure (BiPAP), duration of BiPAP (days), nasal intermittent positive pressure ventilation (NIPPV), duration of NIPPV (days), invasive mechanical ventilation (IMV), duration of IMV (days), noninvasive mechanical ventilation (NIMV), duration of NIMV (days), duration of FiO_2 _> 21% (days), and maximum of FiO_2_ (%). IMV includes CMV and HFV, and NIMV includes CPAP, HFNC, BiPAP and NIPPV.

### Statistical analyses

Statistical analyses were conducted by applying R (v4.1.3) software. Normally distributed continuous variables were displayed as mean (SD) and skewed distributed variables as median (25th, 75th), and Student's *t*-test or Mann-Whitney test were used to compare the two study groups. For categorical variables, frequency (percentage) was displayed and compared using *χ*^2^ test or Fisher exact test.

Based on the significantly different clinical characteristics (*P *< 0.05), we sought to build predictive model for moderate and severe BPD in infants with gestational age <30 weeks and birth weight <1,500 g on postnatal Day 14. First, conforming to a ratio of 3 : 1, we randomly divided the 412 preterm infants into a training set with 309 infants and a validation set with 103 infants. To identify the best predictors of current risk factors, LASSO (least absolute shrinkage and selection operator) regression was performed in the training set using R *glmnet* package (16). As the dependent variable is whether moderate and severe BPD is present or not, we set family = “binomial” and set type.measure = “deviance”. Based on the binomial family and the type measure of deviance, the LASSO regression analysis runs a ten-fold cross-validation for centralizing and normalizing the variables included, and then the best lambda value was picked. In terms of performance, *Lambda.1se* gives the best results, but with the fewest independent variables. Then the R *rms* package was used to run logistic regression to construct a prediction model for moderate and severe BPD by introducing the factors selected in the LASSO regression and the proven risk factors for moderate and severe BPD. All of the selected factors were applied to construct nomogram prediction model. In order to easily approximate the individual-specific risk of moderate and severe BPD in infants with gestational age <30 weeks and birth weight <1,500 g on postnatal Day 14, a graphical nomogram was also created (17).

Additionally, the accuracy of the model was estimated using several validation methods using the data of training set and validation set, respectively (16, 18). Receiver-operator characteristic (ROC) curve was constructed with the R *pROC* package, and the area under the ROC curve (AUC) provided good discrimination between true positives and false positives for the quality of the risk nomogram. Using the “best threshold” criteria of the ROC curve, the sensitivity, specificity, positive predictive value (PPV) and negative predictive value (NPV) were calculated to illustrate the model effects. To evaluate the calibration of the moderate and severe BPD risk nomogram, along with the Hosmer-Lemeshow test, the R *rms* package was used to draw the calibration curves. As well, clinical impact curves were drawn using the R *rmda* package to determine whether the nomogram is clinically practicable for predicting moderate and severe BPD. All statistical significance levels reported were two-sided, and *P *< 0.05 was considered to be significant.

## Results

### Clinical characteristics and treatment

**Figure 1:**
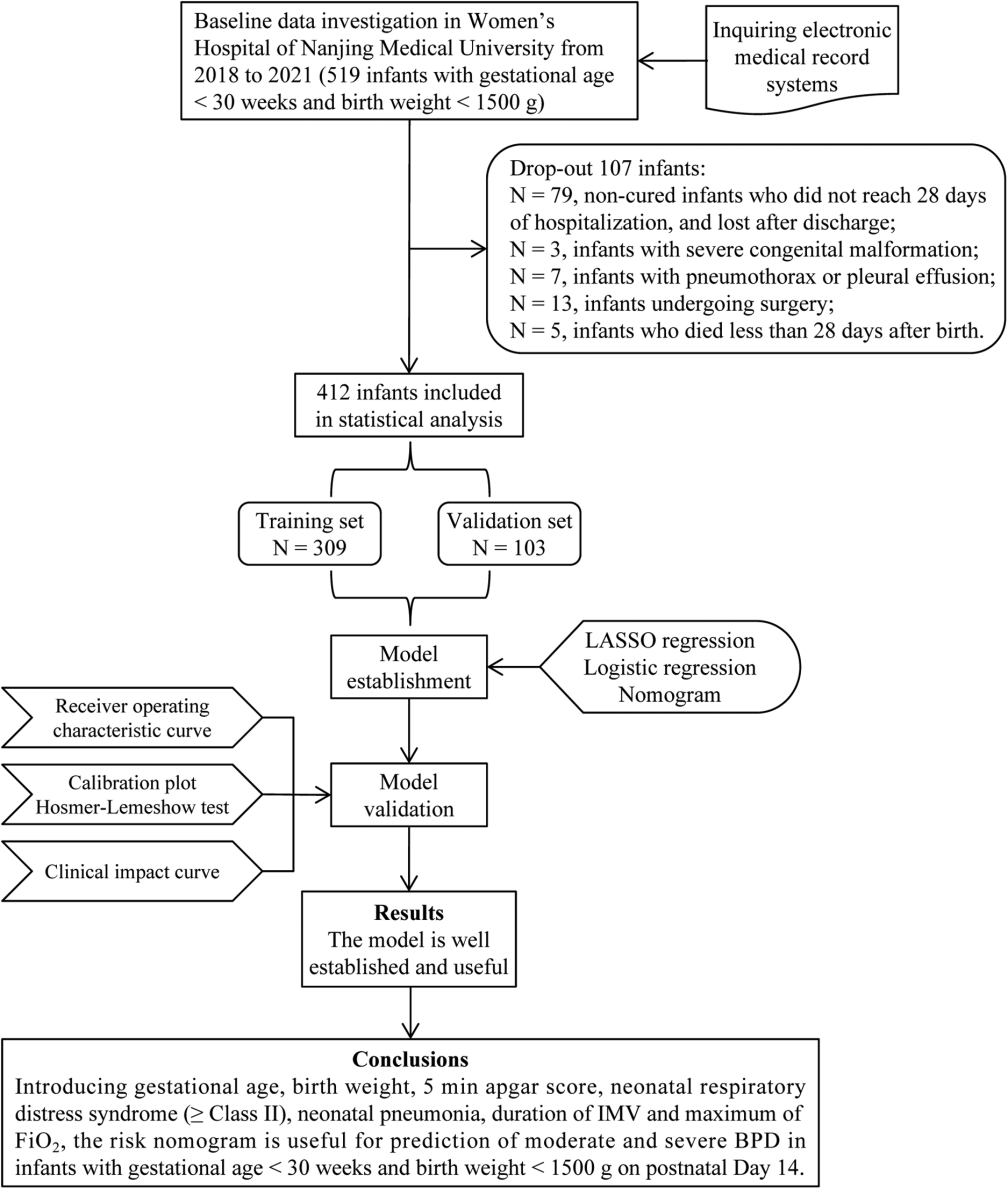
Flow diagram of study design. BPD, bronchopulmonary dysplasia; IMV, invasive mechanical ventilation; FiO_2_, fraction of inspiration O_2_.

In total, 519 infants with gestational age <30 weeks and birth weight <1,500 g were recruited in Nanjing Maternity and Child Health Care Hospital from 2018 to 2021. We excluded 79 uncured infants hospitalized for less than 28 days and lost to follow-up after discharge; 3 infants with severe congenital malformation; 7 infants with pneumothorax or pleural effusion; 13 infants undergoing surgery; and 5 infants who died less than 28 days after birth. At last, 412 infants were included in the analysis (Figure 1), including 104 infants with moderate and severe BPD and 308 infants without moderate and severe BPD (as controls). These infants were randomly divided into a training set with 309 infants and a validation set with 103 infants for external validation.

The maternal characteristics, medication use, neonatal characteristics, mechanical ventilation and oxygen requirement up to postnatal Day 14 are shown in Table 1. In terms of maternal characteristics and medication use, the rates of gestational diabetes mellitus, hypertension, chorioamnionitis, prenatal use of glucocorticoids and magnesium sulfate were similar between the two study groups. In terms of neonatal characteristics, the gestational age, birth weight, cesarean delivery rate, 1 min and 5 min apgar score were significantly lower in infants with moderate and severe BPD than those in control group (all *P *< 0.001). Whereas the rates of neonatal asphyxia, neonatal respiratory distress syndrome (≥Class II), neonatal pneumonia, PDA, PDA treatment, IVH III/IV, sepsis, and NEC (≥Stage II) were significantly higher in infants with moderate and severe BPD (all *P *< 0.05), and the PDA size was also larger in infants with moderate and severe BPD (*P *= 0.002).

We then focused on the characteristics of mechanical ventilation and oxygen requirement up to postnatal Day 14 between the two study groups. In infants with moderate and severe BPD, the rates of CMV, HFV, NIPPV and IMV, and the duration of CMV, HFV, NIPPV and IMV were significantly higher than those in control group (all *P *< 0.01), whereas the rates of CPAP, HFNC and NIMV, and the duration of CPAP, HFNC and NIMV were significantly lower than that in control group (*P *< 0.001). For oxygen requirement, the maximum of FiO_2_ was significantly higher in infants with moderate and severe BPD (*P *< 0.001).

### Construction of predictive model

**Figure 2:**
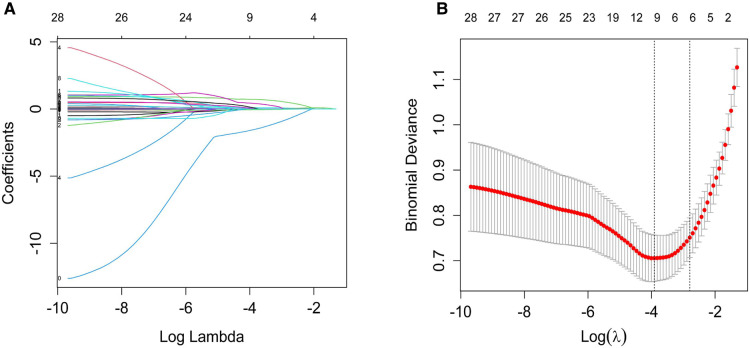
Variable selection by the LASSO binary logistic regression model. (**A**) A coefficient profile plot was constructed against the log(lambda) sequence. Twenty-eight variables with nonzero coefficients were selected by deriving the optimal lambda. (**B**) Following verification of the optimal lambda in the LASSO model, we plotted the binomial deviance curve versus log(lambda) and drew dotted vertical lines based on 1 standard error criteria. LASSO, Least absolute shrinkage and selection operator.

**Figure 3:**
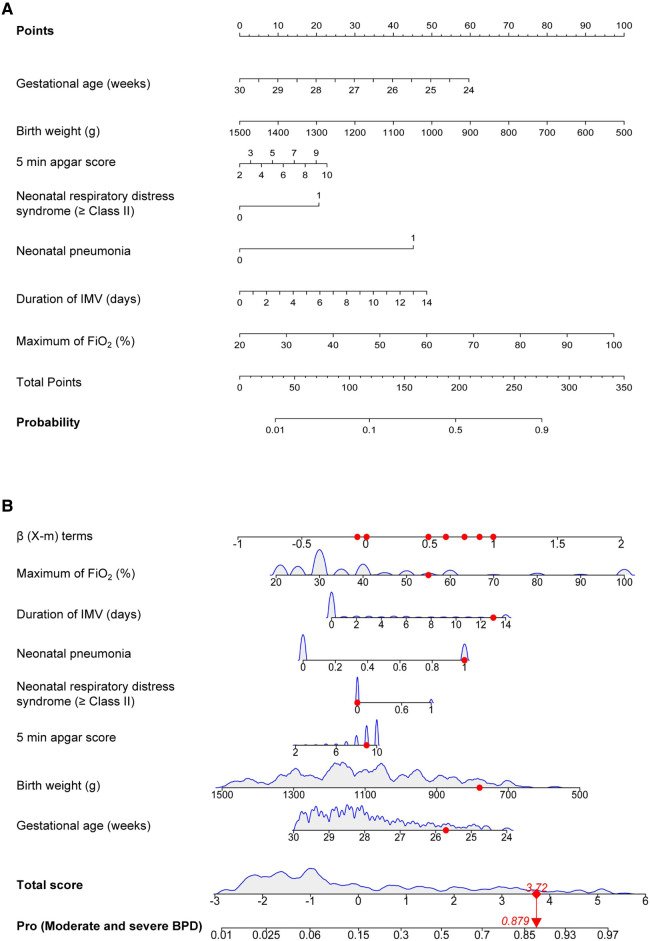
Moderate and severe BPD risk nomogram prediction model. (**A**) Risk factors of gestational age, birth weight, 5 min apgar score, neonatal respiratory distress syndrome (≥Class II), neonatal pneumonia, duration of IMV and maximum of FiO_2_ for nomogram prediction model. (**B**) Dynamic nomogram used as an example. BPD, bronchopulmonary dysplasia; IMV, invasive mechanical ventilation; FiO_2_, fraction of inspiration O_2_.

The significant items in Table 1 were selected for predictive variables using LASSO regression analysis. As a result, 6 of the original 28 variables were considered in the predictive model, including birth weight, 5 min apgar score, neonatal respiratory distress syndrome (≥Class II), neonatal pneumonia, duration of IMV and maximum of FiO_2_ (Figure 2). In the LASSO regression model, these 6 variables had non-zero coefficients. As smaller gestational age is proven risk factors for moderate and severe BPD, we also introduced this variable into the predictive model. In Table 2, we present the results of the logistic regression analysis for these 7 variables. Based on the predictive model, a nomogram was used to quantitatively predict the risk probability of moderate and severe BPD in infants with gestational age <30 weeks and birth weight <1,500 g on postnatal Day 14 (Figure 3A). As an example, an infant with gestational age of 25.7 weeks, birth weight of 780 g, 5 min apgar score of 9, without neonatal respiratory distress syndrome (≥Class II), with neonatal pneumonia, a duration of IMV for 13 days, and maximum of FiO_2_ of 55%, has an estimated probability of moderate and severe BPD of 0.879 (Figure 3B).

**Table 2:** Logistic regression analysis of the predictors for the risk of moderate and severe bronchopulmonary dysplasia (BPD) in infants with gestational age <30 weeks and birth weight <1,500 g on postnatal Day 14.

Intercept and variables	Estimate	Standard error	*z* value	*P* value	Odds ratio	Confidence interval (2.5%)	Confidence interval (97.5%)
Intercept	1.843	3.646	0.506	0.613	6.316	–	–
Gestational age (weeks)	0.002	0.147	0.010	0.992	1.002	0.750	1.337
Birth weight (g)	−0.003	0.001	−2.817	0.005	0.997	0.995	0.999
5 min apgar score	−0.217	0.107	−2.034	0.042	0.805	0.653	0.992
Neonatal respiratory distress syndrome (≥Class II)	1.224	0.405	3.018	0.003	3.400	1.536	7.527
Neonatal pneumonia	1.325	0.324	4.093	<0.001	3.762	1.995	7.097
Duration of IMV (days)	0.116	0.042	2.728	0.006	1.123	1.033	1.22
Maximum of FiO_2_ (%)	0.020	0.009	2.302	0.021	1.020	1.003	1.038

Note: IMV, invasive mechanical ventilation; FiO_2_, fraction of inspiration O_2_.

### Validation of predictive model

**Figure 4:**
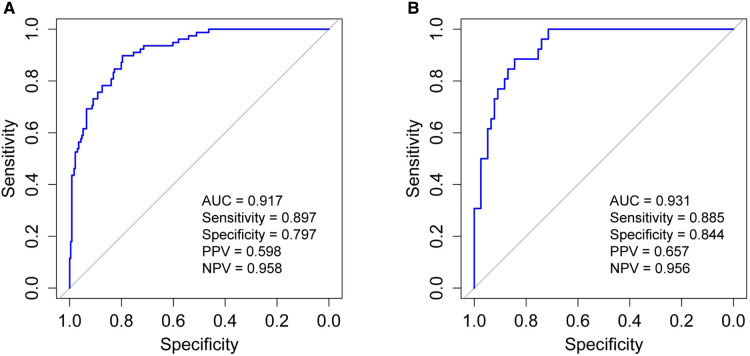
Receiver operating characteristic curve (ROC) validation of the moderate and severe BPD risk nomogram prediction. The *y*-axis represents the true positive rate of the risk prediction, the *x*-axis represents the false positive rate of the risk prediction. (**A**) The performance in the training set; (**B**) the performance in the validation set. BPD, bronchopulmonary dysplasia; AUC, area under the ROC curve; PPV, positive predictive value; NPV, negative predictive value.

**Figure 5:**
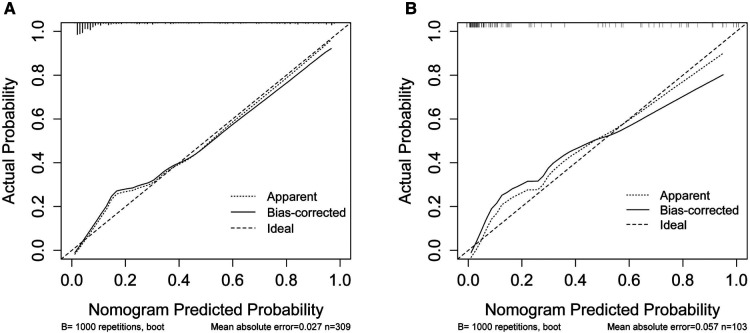
Calibration curves of the moderate and severe BPD risk nomogram. The *y*-axis represents actual diagnosed cases of moderate and severe BPD, the *x*-axis represents the predicted risk of moderate and severe BPD. The diagonal dotted line represents a perfect prediction by an ideal model, the solid line represents the performance of the moderate and severe BPD risk nomogram in the training set (**A**) and validation set (**B**), with the results indicating that a closer fit to the diagonal dotted line represents a better prediction. BPD, bronchopulmonary dysplasia.

**Figure 6:**
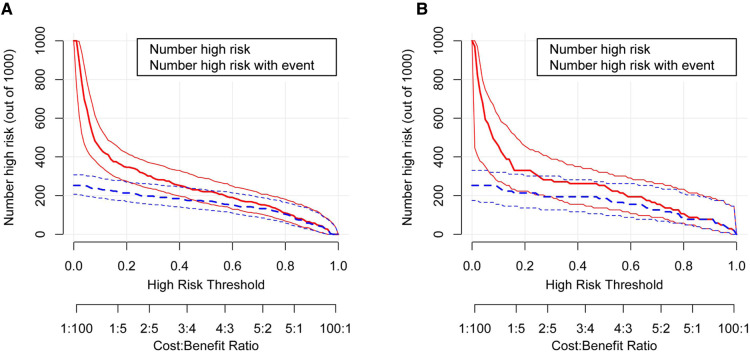
The clinical impact curve for the moderate and severe BPD predictive model. Of 1,000 patients, the red solid line shows the total number who would be deemed high risk for each risk threshold. The blue dashed line shows how many of those would be true positives (cases). (**A**) From the training set; (**B**) from the validation set. BPD, bronchopulmonary dysplasia.

In order to evaluate the predictive model's discriminatory capacity, the ROC curve was used. The pooled AUC of the predictive model is 0.917 (sensitivity = 0.897, specificity = 0.797) in the training set and 0.931 (sensitivity = 0.885, specificity = 0.844) in the validation set, which indicates good performance (Figure 4). We also conducted a sensitivity analysis for severe BPD alone, with the pooled AUC of 0.933 (sensitivity = 0.900, specificity = 0.847) in the total data set. Afterwards, the predictive model was calibrated using a calibration plot and Hosmer–Lemeshow test. According to the calibration curves, the predictive model fit the data very well (Figure 5). Moreover, the Hosmer–Lemeshow test showed high consistency between actual and predicted probabilities (*P *= 0.727 for training set, *P *= 0.809 for validation set). Figure 6 shows the predictive model's clinical impact curves. The red solid line shows the total number of patients deemed high risk at each risk threshold out of 1,000. The blue dashed line indicates how many of those were true positives.

## Discussion

In the prediction model we constructed for moderate and severe BPD, seven variables, namely gestational age, birth weight, 5 min apgar score, neonatal respiratory distress syndrome (≥Class II), neonatal pneumonia, duration of IMV and maximum of FiO_2_, are important predictors for moderate and severe BPD in infants with gestational age <30 weeks and birth weight <1,500 g on postnatal Day 14. Using these 7 predictors, the constructed model displayed good prediction performance for moderate and severe BPD, with an AUC of 0.917 in the training set and 0.931 in the validation set, and was well calibrated. Further sensitivity analysis for severe BPD alone showed the pooled AUC of the model is 0.933 in the total data set. Previously identified risk factors for BPD include gestational age, birth weight, oxygen therapy, mechanical ventilation, and duration of assisted ventilation (12). These factors measured up to postnatal Day 14 were included in our model. Besides, we added 5 min apgar score, neonatal respiratory distress syndrome (≥Class II) and neonatal pneumonia into our model, which have improved the prediction performance for the risk of moderate and severe BPD. Risk factors that were reported to be significant in previous studies but that were not included in our final model include male sex, PDA, NEC, and sepsis (12). These factors were considered in this study and were significantly different between the two study groups, but were not selected by further LASSO regression. Medical resource and management practices for preterm infants, as well as risk factors for BPD, vary from region to region, which may partly explain the differences between models.

In this study, a novel statistical method (LASSO regression) was used to identify the predictors for moderate and severe BPD. LASSO regression analysis minimizes the prediction error of quantitative response variables by imposing constraints on model parameters and reducing the regression coefficients of some variables to zero (16), thus providing more accurate results. A graphical nomogram was produced for obstetricians to easily use the constructed model to quantitatively predict the risk probability of moderate and severe BPD in preterm infants. Moreover, ROC, calibration and clinical impact curves were constructed to verify the model’s stability and accuracy. However, the limitations of this study need to be acknowledged. Firstly, this is a retrospective study. Secondly, due to the limited infants with gestational age <30 weeks and birth weight <1,500 g, the sample size of the training set and validation set was relatively small. Researchers will investigate more thoroughly in the future by involving a larger sample of preterm infants and assessing a broader range of risk factors.

In the comparison of mechanical ventilation characteristics and oxygen requirement between the two study groups, we found that duration of IMV, duration of FiO_2 _> 21%, and maximum of FiO_2_ in the infants with moderate and severe BPD were significantly higher than those in the control group, which was consistent with most studies (19, 20). Mechanical ventilation and high concentration of oxygen can cause increased reactive oxygen species and alveolar hyper-expansion, resulting in local oxidative stress injury, pressure and volume injury (20, 21). Interestingly, only duration of IMV and maximum of FiO_2_ were identified in further LASSO regression analysis. It is estimated that 65% of the preterm infants with birth weight <1,500 g received IMV support in the delivery room and/or during their admission (22). Although IMV can be lifesaving and improve the preterm infants’ respiratory status, it is related with increased risks of BPD, air leak syndrome and neurodevelopmental impairment, and may result in long-lasting consequences (20, 23). To reduce these adverse effects of IMV, preterm infants are increasingly received non-invasive respiratory support, often beginning in the delivery room (22). In general, NIMV refers to any ventilator support technique that does not require tracheal intubation but uses constant or variable pressure. In modern neonatal ventilators, improvements in the measurement of flow and volume have led to a variety of alternative NIMV procedures, such as CPAP, HFNC, BiPAP and NIPPV. In clinical practice, restricting IMV usage has been shown to be feasible and reduce the incidence of BPD and neurodevelopmental impairments (19, 24, 25). In addition, risk for respiratory disease has often been quantified by duration and concentration of supplemental oxygen, both of which could contribute to oxygen toxicity and serve as a marker for severity of disease (21, 26). Cumulative supplemental oxygen has been shown to be independently associated with BPD or death (21). In this study, based on the clinical characteristics and the different types of mechanical ventilation, we developed a meaningful risk prediction model for paediatricians and neonatologists to perform early screening of moderate and severe BPD in infants with gestational age <30 weeks and birth weight <1,500 g on postnatal Day 14. The prediction model could be used to target VLBWI at the highest risk of moderate and severe BPD, to tailor follow-up and preventive measures for VLBWI.

## Conclusions

The 7 predictors verified by nomogram, including gestational age, birth weight, 5 min apgar score, neonatal respiratory distress syndrome (≥Class II), neonatal pneumonia, duration of IMV and maximum of FiO_2_, are very meaningful in identifying risk of moderate and severe BPD in infants with gestational age <30 weeks and birth weight <1,500 g on postnatal Day 14. Also, these indicators are helpful for early screening of moderate and severe BPD and provide prognostic information for families and clinicians.

## Data Availability

The original contributions presented in the study are included in the article/Supplementary Material, further inquiries can be directed to the corresponding author/s.
